# Early mortality during rifampicin-resistant TB treatment

**DOI:** 10.5588/ijtld.21.0494

**Published:** 2022-02-01

**Authors:** E. Mohr-Holland, J. Daniels, A. Reuter, C. A. Rodriguez, C. Mitnick, Y. Kock, V. Cox, J. Furin, H. Cox

**Affiliations:** 1 Khayelitsha Project, Médecins Sans Frontières (MSF), Cape Town, South Africa; 2 Southern Africa Medical Unit, MSF, Cape Town, South Africa; 3 Department of Global Health and Social Medicine, Harvard Medical School, Boston, MA, USA; 4 National Department of Health, Pretoria, South Africa; 5 Center for Infectious Disease Epidemiology and Research, School of Public Health and Family Medicine, University of Cape Town, Cape Town, South Africa; 6 Division of Medical Microbiology, Department of Pathology, University of Cape Town, Cape Town, South Africa; 7 Institute for Infectious Disease and Molecular Medicine and Wellcome Centre for Infectious Disease Research in Africa, University of Cape Town, Cape Town, South Africa

**Keywords:** drug-resistant tuberculosis, death, bedaquiline, linezolid, delamanid

## Abstract

**BACK GROUND:**

Data suggest that treatment with newer TB drugs (linezolid [LZD], bedaquiline [BDQ] and delamanid [DLM]), used in Khayelitsha, South Africa, since 2012, reduces mortality due to rifampicin-resistant TB (RR-TB).

**METHODS:**

This was a retrospective cohort study to assess 6-month mortality among RR-TB patients diagnosed between 2008 and 2019.

**RESULTS:**

By 6 months, 236/2,008 (12%) patients died; 12% (78/651) among those diagnosed in 2008–2011, and respectively 8% (49/619) and 15% (109/738) with and without LZD/BDQ/DLM in 2012–2019. Multivariable analysis showed a small, non-significant mortality reduction with LZD/BDQ/DLM use compared to the 2008–2011 period (aOR 0.79, 95% CI 0.5–1.2). Inpatient treatment initiation (aOR 3.2, 95% CI 2.4–4.4), fluoroquinolone (FQ) resistance (aOR 2.7, 95% CI 1.8–4.2) and female sex (aOR 1.5, 95% CI 1.1–2.0) were also associated with mortality. When restricted to 2012–2019, use of LZD/BDQ/DLM was associated with lower mortality (aOR 0.58, 95% CI 0.39–0.87).

**CONCLUSIONS:**

While LZD/BDQ/DLM reduced 6-month mortality between 2012 and 2019, there was no significant effect overall. These findings may be due to initially restricted LZD/BDQ/DLM use for those with high-level resistance or treatment failure. Additional contributors include increased treatment initiation among individuals who would have otherwise died before treatment due to universal drug susceptibility testing from 2012, an effect that also likely contributed to higher mortality among females (survival through to care-seeking).

## Abstract (fr)

**CONTEXTE:**

Certaines données suggèrent qu’un traitement à base des nouveaux médicaments antituberculeux (linézolide [LZD], bédaquiline [BDQ] et délamanide [DLM]), administré à Khayelitsha, Afrique du Sud, depuis 2012, réduit la mortalité due à la TB résistante à la rifampicine (RR-TB).

**MÉTHODES:**

Il s’agissait d’une étude de cohorte rétrospective visant à évaluer la mortalité à 6 mois chez des patients atteints de RR-TB diagnostiqués entre 2008 et 2019.

**RÉSULTATS:**

À 6 mois, 236/2 008 (12%) patients étaient décédés ; 12% (78/651) de ceux diagnostiqués en 2008–2011, et respectivement 8% (49/619) et 15% (109/738) avec et sans LZD/BDQ/DLM en 2012–2019. L’analyse multivariable a démontré une faible réduction non significative de la mortalité avec l’utilisation de LZD/BDQ/DLM par rapport à la période 2008–2011 (aOR 0,79 ; IC 95% 0,5–1,2). L’instauration du traitement chez les patients hospitalisés (aOR 3,2 ; IC 95% 2,4–4,4), la résistance aux fluoroquinolones (FQ) (aOR 2,7 ; IC 95% 1,8–4,2) et le genre féminin (aOR 1,5 ; IC 95% 1,1–2,0) étaient également associés à la mortalité. En restreignant l’analyse à la période 2012–2019, l’utilisation de LZD/BDQ/DLM était associée à une plus faible mortalité (aOR 0,58 ; IC 95% 0,39–0,87).

**CONCLUSIONS:**

Bien que LZD/BDQ/DLM aient réduit la mortalité à 6 mois entre 2012 et 2019, aucun effet global significatif n’a été observé. Ces résultats peuvent être dus à l’utilisation initiale restreinte de LZD/BDQ/DLM pour les patients présentant de forts taux de résistance ou en échec de traitement. Les facteurs contributifs supplémentaires comprennent une hausse des instaurations de traitement chez des patients qui seraient autrement décédés avant l’instauration en raison de la mise en place universelle des tests de sensibilité aux médicaments à partir de 2012. Cet effet a également probablement contribué à une plus grande mortalité chez les femmes (survie jusqu’à la recherche de soins).

TB remains one of the leading infectious killers globally, and in 2019 alone, 182,000 people are estimated to have died from multidrug/rifampicin-resistant TB (MDR/RR-TB).^1^ Recent advances in the diagnosis and treatment of MDR/RR-TB have the potential to markedly improve treatment success, which was only 57% globally in 2017.^1^ Specifically, the use of several medications—including linezolid (LZD), bedaquiline (BDQ) and third-generation fluoroquinolones (FQs) have been associated with decreased mortality.^2^

South Africa is a global leader in the public health response to MDR/RR-TB with early implementation of new diagnostic tools and treatment regimens. The use of Xpert^®^ MTB/RIF (Cepheid, Sunnyvale, CA, USA), which enabled universal drug susceptibility testing (DST), was initiated in 2011;^3^ early access to LZD/BDQ/delamanid (DLM) for MDR/RR-TB treatment was facilitated by the National Department of Health through clinical access programmes initially, with later scale-up.^4^,^5^ South African data show that the use of LZD/BDQ has improved patient outcomes, including significant reductions in mortality and treatment failure with BDQ-containing regimens.^4^–^9^ Despite these improvements, RR-TB treatment success across South Africa remained at 60% in 2017.^1^ Few data are available to assess the impact of improved access to DST and LZD/BDQ/DLM on patient outcomes over time.

In Khayelitsha, South Africa, a setting with high MDR/RR-TB and HIV burdens, Xpert was rolled out in late 2011 and LZD/BDQ/DLM have been used since 2012, initially for patients meeting certain criteria,^4^,^7^–^10^ and later more generally with changes in national guidance.^11^ Here, we assess changes in mortality since universal access to Xpert and introduction of LZD/BDQ/DLM in Khayelitsha.

## METHODS

### Study design

This was a retrospective cohort analysis assessing 6-month mortality among patients treated for MDR/RR-TB from 2008 to 2019 inclusive. Patients were included if they were bacteriologically confirmed with MDR/RR-TB, started treatment during the study period and had confirmation of their mortality status at 6 months.

### Setting

Khayelitsha is a peri-urban township in Cape Town, South Africa, with approximately 450,000 people.^12^ There are 180–200 patients diagnosed with MDR/RR-TB annually (estimated case notification rate 55/100,000) and rates of HIV co-infection are approximately 70%.^10^,^13^ There are 11 primary health care (PHC) facilities within Khayelitsha offering decentralised MDR/RR-TB treatment and care.^3^,^10^

Criteria for access to LZD/BDQ/DLM has changed over time. Access was initially available to those with second-line (SL) TB drug resistance for whom an adequate regimen could not be constructed or those with MDR/RR-TB treatment failure; this was later expanded to include those requiring drug substitution for any reason.^7^,^9^ Universal access to BDQ-based, all-oral regimens became available from 2018 onward.

### Data collection

A routine programmatic MDR/RR-TB database has been maintained since 2008 in Khayelitsha.^10^ A cohort of treated patients diagnosed for the first time between 1 January 2008 and 31 December 2019 was extracted. Additional mortality data were obtained from the South African vital status registry using national identification numbers. For patients with no available identification number, the presence of any laboratory data was used to confirm if individuals were still alive at 6 months.

### Definitions

As both Xpert and LZD/BDQ/DLM were introduced from 2012 onward, the cohort was divided into three groups: those diagnosed in 2008–2011 and those diagnosed across 2012–2019 divided by receipt of any one of LZD/BDQ/DLM initiated within 6 months of MDR/RR-TB treatment initiation. As clinical status was the primary criteria for treatment initiation in hospital, this was used as a proxy for clinical condition. All-cause mortality was defined as death from any cause within the 6-month follow-up period following MDR/RR-TB treatment initiation, regardless of whether the individuals were receiving treatment at death.

### Data analysis

Mortality at 6 months was stratified by clinical and demographic characteristics. χ^2^ tests and Wilcoxon Signed Rank Tests were used to determine significant differences in categorical and continuous variables, respectively. Logistic regression was used to determine crude and adjusted odds ratios (aORs) and 95% confidence intervals (CIs) for factors associated with 6-month mortality. Clinically relevant factors (sex, age, HIV status and antiretroviral therapy [ART] initiation, site of MDR/RR-TB treatment initiation, previous TB treatment history, and year of diagnosis, and whether LZD/BDQ/DLM were received within 6 months of treatment initiation) and factors with *P-*values <0.2 from the univariable analysis were included in the multivariable analysis. Stata v14 (Stata Corp, College Station, TX, USA) was used for statistical analyses.

We hypothesised that observed associations of sex and mortality may have been driven by systematic under-diagnosis and treatment of males, which would result in the exclusion of males from the programmatic database. To assess the potential impact of this mechanism of survivor selection bias, we calculated the additional number of unobserved deaths that would need to occur in males in order to match the mortality prevalence in females using summary-level quantitative bias analysis.^14^

### Ethics

Ethics approval for this study was obtained from the University of Cape Town Human Research Ethics Committee, Cape Town, South Africa (HREC 540/2010). The study fulfilled the Médecins Sans Frontières Ethics Review Board (Geneva, Switzerland) exemption criteria.

## RESULTS

### Cohort description

Overall, 2,032 bacteriologically confirmed patients were diagnosed and received MDR/RR-TB treatment between 2008 and 2019; 24 (1.2%) patients were excluded, as 6-month mortality status was unavailable. Of the remaining 2,008 patients, 953 (48%) were female, the median age was 34 years, and 1,445 (72%) were HIV-positive with a median CD4 count of 121 cells/mm^3^ (Table 1). Overall, 651 (32%) were diagnosed between 2008 and 2011, 738 (37%) between 2012 and 2019, but did not receive LZD/BDQ/DLM, and 619 (31%) between 2012 and 2019 who received LZD/BDQ/DLM. Patients diagnosed between 2012 and 2019 had a shorter time between MDR/RR-TB diagnosis and both ART and MDR/RR-TB treatment initiation, more frequently initiated treatment in primary care, and were less likely to have been previously treated compared to the earlier time period (*P* < 0.05, data not shown). Patients diagnosed between 2012 and 2019 who received LZD/BDQ/DLM were more likely to have FQ resistance (*n* = 116/630, 18% vs. 36/746, 5%; *P* < 0.001).

**Table 1:** Clinical and demographic characteristics of all patients and patients with mortality at 6 months among those diagnosed and treated for MDR/RR-TB from 2008 to 2019, including univariate and multivariable analyses of factors associated with mortality at 6 months

Factor in univariate analysis	Total (*n* = 2008)	Died (*n* = 236, 11.8%) *n* (%)	Time to death Months Median (IQR)	OR (95% CI)	*P* value	aOR (95% CI)	*P* value
Overall			2.0 (1.0–3.4)				
Sex							
Male	1,055 (52.5)	102 (9.7)	2.2 (1.0–3.9)	1 (reference)	—	1 (reference)	—
Female	953 (47.5)	132 (14.1)	1.9 (1.0–3.3)	1.5 (1.2–2.0)	<0.01^*^	1.5 (1.1–2.0)	0.01^*^
Age, years							
0–24	334 (16.6)	20 (6.0)	1.8 (1.0–4.3)	0.48 (0.3–0.8)	<0.01^*^	0.53 (0.31–0.91)	0.02^*^
25–39	1,040 (51.8)	122 (11.7)	2.2 (1.0–3.8)	1 (reference)	—	1 (reference)	—
≥40	634 (31.6)	94 (14.8)	1.9 (0.9–3.0)	1.3 (1.0–1.7)	0.07^*^	1.5 (1.1–2.1)	<0.01^*^
Year of diagnosis							
2008–2011	651 (32.4)	78 (12.0)	2.4 (1.0–4.0)	1 (reference)	—	1 (reference)	––
2012–2019, no LZD/BDQ/DLM received	738 (36.8)	109 (14.8)	1.6 (0.7–2.8)	1.3 (0.9–1.7)	0.13^*^	1.5 (1.0–2.1)	0.03^*^
2012–2019, LZD/BDQ/DLM received	619 (30.8)	49 (7.9)	2.5 (1.7–4.0)	0.63 (0.43–0.92)	0.02^*^	0.79 (0.5–1.2)	0.26
HIV status and started ART							
HIV-negative	563 (28.0)	34 (6.0)	2.7 (1.3–4.1)	1 (reference)	––	1 (reference)	––
HIV-positive, on ART	1,351 (67.3)	154 (11.4)	2.2 (1.2–3.9)	2.0 (1.4–2.9)	<0.01^*^	1.6 (1.0–2.4)	0.04^*^
HIV-positive, no ART	94 (4.7)	48 (51.5)	1.0 (0.3–2.1)	16.2 (9.5–27.7)	<0.01^*^	12.6 (7.0–22.4)	<0.01^*^
Site of treatment initiation							
Outpatient	1,543 (76.8)	125 (8.1)	2.3 (1.2–4.0)	1 (reference)	––	1 (reference)	––
In patient	465 (23.2)	111 (23.9)	1.9 (0.8–3.0)	3.6 (2.7–4.7)	<0.01^*^	3.2 (2.4–4.4)	<0.01^*^
Resistance classification							
RR-TB^†^	550 (27.4)	64 (11.6)	2.1 (0.9–4.0)	1.2 (0.9–1.6)	0.33	0.9 (0.6–1.3)	0.76
MDR-TB^‡^	1,256 (62.6)	127 (10.1)	2.1 (1.0–3.4)	1 (reference)	––	1 (reference)	—
FQ resistance	202 (10.1)	45 (22.3)	1.7 (1.0–3.2)	2.5 (1.7–3.7)	<0.01^*^	2.7 (1.8–4.2)	<0.01^*^
Previous TB treatment history							
None	694 (34.6)	72 (10.4)	1.8 (0.7–2.9)	1 (reference)	––	1 (reference)	––
Previous first-line TB treatment	1,312 (65.3)	164 (12.5)	2.2 (1.1–4.0)	1.2 (0.9–1.7)	0.16^*^	1.1 (0.8–1.5)	0.63
Unknown	2 (0.1)	0 (0.0)	––	––	––	–	–

* Statistically significant (*P* < 0.2 and *P* <0.05 were considered significant in univariate analysis and multivariate analysis, respectively).

^†^Includes those with RIF resistance detected using Xpert MTB/RIF without culture confirmation and those with confirmed RIF monoresistance.

^‡^Includes those with resistance to RIF and isoniazid, including resistance to the injectable agent.

MDR-TB =multidrug-resistant TB; RR-TB=RIF-resistant TB; OR=odds ratio; CI=confidence interval; aOR=adjusted OR; LZD=linezolid; BDQ=bedaquiline; DLM=delamanid; ART = antiretroviral therapy; FQ = fluoroquinolone; RIF = rifampicin.

### 6-month mortality

By 6 months, 236 (12%) patients died; of these, respectively 13 (6%) and 7 (3%) were no longer receiving treatment at the time of death as they were either lost to follow-up (LTFU) or transferred out. Overall, mortality was lowest among patients diagnosed in 2012–2019 who received LZD/BDQ/DLM, 8%, compared to respectively 12% and 15% of those diagnosed in 2008–2011 and 2012–2019 without LZD/BDQ/DLM (Figure).

### Factors associated with 6-month mortality

In univariable logistic regression analysis, female sex, HIV positivity regardless of ART status, initiation of MDR/RR-TB treatment as an inpatient and FQ resistance were associated with higher mortality, while younger age and use of LZD/BDQ/DLM with diagnosis and treatment in 2012–2019 were protective (Table 1).

In multivariable analysis, higher mortality was associated with diagnosis in 2012–2019 without use of LZD/BDQ/DLM, HIV positivity regardless of ART status, initiation of MDR/RR-TB treatment as an inpatient and FQ resistance (Table 1). Furthermore, female sex was associated with a 50% mortality increase (adjusted OR [aOR] 1.5, 95% CI 1.1–2.0), while younger age (0–24 years) was protective (Table 1).

As mortality significantly differed between those who did and did not receive LZD/BDQ/DLM and diagnosed in 2012–2019, logistic regression analyses were conducted for these groups separately and among the 2012–2019 group overall. Among all patients diagnosed in 2012–2019, age ≥40 years, HIV positivity and no ART, inpatient treatment initiation and FQ resistance were associated with increased mortality (Table 2). Conversely, receipt of LZD/BDQ/DLM was protective (aOR 0.58, 95% CI 0.39–0.87), as was younger age (Table 2). It is surprising to note that mortality was higher among females (aOR 1.6, 95% CI 1.1–2.4; Table 2). This remained significant for females who did not receive LZD/BDQ/DLM (aOR 2.0, 95% CI 1.2–3.2; Table 3A); however, among those who received LZD/BDQ/DLM there was no significant difference (aOR 1.1, 95% CI 0.6–2.1; Table 3B).

**Table 2:** Logistic regression analysis of factors associated with mortality among those diagnosed 2012–2019 who were initiated on treatment (n = 1357)

Factor	Died (*n* = 158, 11.6) *n* (%)	Median time to death (months)	OR (95% CI)	*P* value	aOR (95% CI)	*P* value
Overall		2.5 (1.7–4.0)				
Sex						
Male	67 (9.3)	2.1 (1.1–3.9)	1 (reference)	—	1 (reference)	—
Female	91 (14.4)	1.9 (0.9–3.0)	1.6 (1.2–2.3)	<0.01^*^	1.6 (1.1–2.4)	0.01^*^
Age, years						
0–24	11 (5.1)	1.9 (0.7–3.3)	0.42 (0.2–0.8)	<0.01^*^	0.4 (0.2–0.9)	0.02^*^
25–39	78 (11.4)	2.0 (1.0–3.2)	1 (reference)	—	1 (reference)	—
≥40	69 (15.1)	1.7 (0.9–3.0)	1.4 (1.0–1.9)	0.07^*^	1.6 (1.1–2.4)	0.02^*^
HIV status and started ART						
HIV-negative	21(5.5)	2.7 (1.8–4.0)	1 (reference)	––	1 (reference)	—
HIV-positive, on ART	104 (11.3)	2.1 (1.1–3.3)	2.2 (1.3–3.6)	<0.01^*^	1.5 (0.9–2.6)	0.10
HIV-positive, no ART	33 (60.0)	0.9 (0.2–1.8)	25.8 (12.9–51.7)	<0.01^*^	17.0 (8.0–36.0)	<0.01^*^
Site of treatment initiation						
Outpatient	89 (8.1)	2.0 (1.2–3.4)	1 (reference)	––	1 (reference)	—
In patient	69 (26.4)	1.6 (0.9–2.8)	4.1 (2.9–5.8)	<0.01^*^	3.3 (2.3–4.9)	<0.01^*^
Resistance classification						
RIF resistance^†^	53 (12.7)	1.8 (0.7–3.0)	1.3 (0.9–1.9)	0.14^*^	1.0 (0.7–1.5)	0.95
Multidrug resistance^‡^	78 (9.9)	2.0 (1.0–3.3)	1 (reference)	––	1 (reference)	—
FQ resistance	27 (17.9)	1.8 (1.0–3.2)	2.0 (1.2–3.2)	<0.01^*^	2.1 (1.2–3.7)	<0.01^*^
Previous TB treatment history						
None	54 (9.9)	1.6 (0.6–2.7)	1 (reference)	––––	1 (reference)	—
Previous first-line TB treatment	104 (12.9)	2.1 (1.1–3.4)	1.3 (0.9–1.9)	0.10^*^	1.1 (0.8–1.7)	0.54
LZD/BDQ/DLM received within 6 months of treatment initiation						
No	109 (14.8)	1.4 (0.7–2.8)	1 (reference)	––	1 (reference)	—
Yes	49 (7.9)	2.5 (1.7–4.0)	0.50 (0.3–0.7)	<0.01^*^	0.58 (0.39–0.87)	<0.01^*^

^*^ Statistically significant (*P* < 0.2 and *P* <0.05 were considered significant in univariate analysis and multivariate analysis, respectively).

^†^Includes those with RIF resistance detected using Xpert MTB/RIF without culture confirmation and those with confirmed RIF monoresistance.

^‡^Includes those with resistance to RIF and isoniazid, including resistance to the injectable agent.

OR = odds ratio; CI = confidence interval; aOR = adjusted OR; ART = antiretroviral therapy; RIF = rifampicin; FQ = fluoroquinolone; LZD = linezolid; BDQ = bedaquiline; DLM = delamanid.

**Table 3:** Logistic regression analysis of factors associated among those diagnosed in 2012–2019 who were initiated on treatment regimens which did not include (n = 738) and included (n = 619) LZD/BDQ/DLM

Factor	No LZD/BDQ/DLM		Receipt of LZD/BDQ/DLM	
	
	aOR (95% CI)	*P* value	aOR (95% CI)	*P* value
Sex				
Male	1 (reference)	––	1 (reference)	––
Female	2.0 (1.2–3.2)	<0.01^*^	1.1 (0.6–2.1)	0.72
Age, years				
0–24	0.3 (0.1–0.8)	0.02^*^	0.8 (0.2–2.6)	0.70
25–39	1 (reference)	––	1 (reference)	––
≥40	1.5 (0.9–2.5)	0.09^*^	1.9 (1.0–3.8)	0.05^*^
HIV status and started ART				
HIV-negative	1 (reference)	––	1 (reference)	––
HIV-positive, on ART	1.6 (0.8–3.1)	0.21	1.6 (0.7–3.6)	0.28
HIV-positive, no ART	18.8 (7.6–46.5)	<0.01^*^	11.9 (2.5–58.2)	<0.01^*^
Site of treatment initiation				
Outpatient	1 (reference)	––	1 (reference)	––
Inpatient	3.1 (1.9–5.0)	<0.01^*^	4.1 (2.1–8.0)	<0.01^*^
Resistance classification				
RIF resistance^†^	0.9 (0.5–1.4)	0.59	0.10 (0.7–3.1)	0.26
Multidrug resistance^‡^	1 (reference)	––	1 (reference)	––
FQ resistance	3.4 (1.4–7.8)	<0.01^*^	1.7 (0.8–3.7)	0.19
Previous TB treatment history				
None	1 (reference)	––	1 (reference)	––
Previous first-line TB treatment	1.1 (0.7–1.8)	0.70	1.3 (0.7–2.6)	0.41

* Statistically significant (*P* < 0.2 and *P* <0.05 were considered significant in univariate analysis and multivariate analysis, respectively).

^†^Includes those with RIF resistance detected using Xpert MTB/RIF without culture confirmation and those with confirmed RIF monoresistance.

^‡^Includes those with resistance to RIF and isoniazid, including resistance to the injectable agent.

LZD = linezolid; BDQ = bedaquiline; DLM = delamanid; aOR = adjusted odds ratio; CI = confidence interval; ART = antiretroviral therapy; RIF = rifampicin; FQ = fluoroquinolone.

**Figure:**
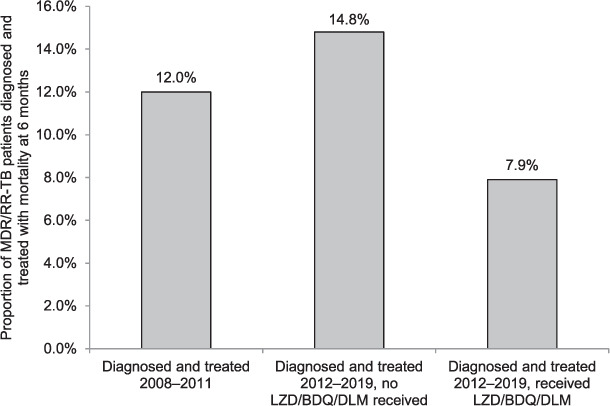
Mortality 6 months after MDR/RR-TB treatment initiation among those diagnosed and initiated on treatment in Khayelitsha, South Africa, 2008–2019 based on year of diagnosis and receipt of LZD/BDQ/DLM; P = 0.13 for comparison of 2008–2011 vs. 2012–2019, no LZD/BDQ/DLM; and P < 0.001 for comparison of 2012–2019 who did and did not receive LZD/BDQ/DLM. MDR/RR-TB = multidrug-/rifampicin-resistant TB; LZD = linezolid; BDQ = bedaquiline; DLM = delamanid.

### Differences in 6-month mortality between males and females

Given the increased mortality among female patients, we investigated differences in baseline clinical and demographic characteristics. Females were more likely to be younger (32 vs. 36 years, *P* < 0.001), HIV-positive (80% vs. 65%, *P* < 0.001) and initiated on MDR/RR-TB treatment as an inpatient (27% vs. 20%, *P* < 0.001). Furthermore, FQ resistance was higher among females than males (11.9% vs. 8.4%, *P*=0.011). There was no difference in the proportion of males and females who received LZD/BDQ/DLM (32% vs. 29%, *P* > 0.05).

In a separate multivariable analysis among females diagnosed in 2012–2019, FQ resistance was significantly associated with increased mortality, while receipt of LZD/BDQ/DLM was protective (aOR 0.5, 95% CI 0.3–0.8). Among males diagnosed in the same period, there was no significant effect of LZD/BDQ/DLM (aOR 0.8, 95% CI 0.5–1.4; Table 4A and B).

**Table 4:** Logistic regression analysis of factors associated with mortality among males (n = 723) and females (n = 634) diagnosed in 2012–2019 who were initiated on treatment

Factor	Males, multivariable		Females, multivariable	
	
	aOR (95% CI)	*P* value	aOR (95% CI)	*P* value
Age, years				
0–24	0.9 (0.3–2.5)	0.77	0.2 (0.1–0.7)	0.01^*^
25–39	1 (reference)	—	1 (reference)	—
≥40	1.5 (0.9–2.7)	0.13	1.6 (0.9–2.8)	0.11
HIV status and started ART				
HIV-negative	1 (reference)	—	1 (reference)	—
HIV-positive, on ART	1.5 (0.7–2.9)	0.30	1.9 (0.8–4.5)	0.14
HIV-positive, no ART	10.7 (4.1–27.8)	<0.01^*^	49.0 (12.0–200.5)	<0.01^*^
Site of treatment initiation				
Outpatient	1 (reference)	—	1 (reference)	—
Inpatient	2.1 (1.2–4.0)	0.01^*^	5.0 (2.9–8.5)	<0.01^*^
Resistance classification				
RIF resistance^†^	1.4 (0.8–2.5)	0.23	0.7 (0.4–1.4)	0.35
Multidrug resistance^‡^	1 (reference)	—	1 (reference)	—
FQ resistance	1.4 (0.6–3.5)	0.43	2.9 (1.4–6.0)	<0.01^*^
Previous TB treatment history				
None	1 (reference)	—	1 (reference)	—
Previous first-line TB treatment	1.5 (0.8–2.8)	0.19	1.0 (0.6–1.8)	1.00
LZD/BDQ/DLM received within 6 months of treatment initiation				
No	1 (reference)	—	1 (reference)	—
Yes	0.8 (0.5–1.4)	0.48	0.5 (0.3–0.8)	0.01^*^

*Statistically significant (*P* < 0.2 and *P* <0.05 were considered significant in univariate analysis and multivariate analysis, respectively).

^†^Includes those with RIF resistance detected using Xpert MTB/RIF without culture confirmation and those with confirmed RIF monoresistance.

^‡^Includes those with resistance to RIF and isoniazid, including resistance to the injectable agent. aOR =adjusted odds ratio; CI=confidence interval; ART=antiretroviral therapy; RIF=rifampicin; FQ=fluoroquinolone; LZD = linezolid; BDQ = bedaquiline; DLM = delamanid.

### Quantitative bias analysis

For the observed OR of mortality among females diagnosed in 2008–2019 (OR 1.5, 95% CI 1.1–2.0) to be due entirely to a survivor selection bias among males (i.e., more males died before diagnosis and treatment initiation), there would need to be 53 additional males who died (i.e., 155 deaths rather than 102 deaths) prior to diagnosis and treatment who were consequently not captured in the programmatic database. If 100% of female deaths were captured, this represents a 34% under-ascertainment of deaths among males. Similarly, across 2012–2019, regardless of the receipt of LZD/BDQ/DLM, an additional 43 male deaths would be needed to match the mortality prevalence of females, representing a 39% under-ascertainment of deaths among males.

## DISCUSSION

Here, we investigated factors associated with early 6-month mortality among patients diagnosed and treated for MDR/RR-TB from 2008 to 2019 in Khayelitsha to assess the impact of improved diagnostics and LZD/BDQ/DLM. While there was an overall reduction in mortality among patients treated with LZD/BDQ/DLM in the 2012–2019 cohort, in adjusted analysis across the whole cohort there was only a small, non-significant reduction in mortality with the use of LZD/BDQ/DLM. As expected, key factors associated with mortality were HIV positivity with no ART,^15^ inpatient treatment initiation,^16^ FQ resistance^17^ and older age.^16^

The mortality benefit of LZD/BDQ/DLM across 2008–2019 may have been lessened by several factors; initially BDQ use was limited to 6 months and there was gradual inclusion of HIV-positive individuals with CD4 <100 cells/mm^3^. Also, in the earlier time period, delayed MDR/RR-TB diagnosis due to restricted DST access most likely contributed to a survivor effect in the 2008–2011 cohort, with consequent lower early mortality. Finally, access to DLM for FQ-resistant patients was only initiated gradually from 2018.

Across the whole cohort, FQ resistance was associated with significantly higher early mortality. However, our findings suggest that FQ resistance can be overcome by the use of LZD/BDQ/DLM; FQ resistance was not significantly associated with early mortality among patients who received LZD/BDQ/DLM, but was a significant predictor in those who did not. These findings highlight the need for rapid implementation of global guidance on the use of new, all-oral regimens that include LZD/BDQ/DLM for MDR/RR-TB treatment,^18^ particularly in settings with significant FQ resistance.

An unexpected finding in this study was the 1.5–2 fold higher early mortality among females compared to males. This difference was more pronounced among females diagnosed in 2012–2019 who did not receive LZD/BDQ/DLM. This is in contrast to most studies which suggest males are more likely to experience death or poor outcomes.^19^,^20^ However, some studies show higher mortality among females on treatment for TB and MDR/RR-TB.^15^,^21^–^23^ Indeed, a recent large-scale analysis of mortality on TB treatment across South Africa shows that women aged 15–24 years had the highest mortality.^23^ Berrut et al. suggested that increased mortality among females on TB treatment might be a result of underreported TB mortality in men, as high as twofold, in developing countries.^22^

Findings from the quantitative bias analysis suggest that a third of deaths among males would need to be missed for the survivor effect to explain all the sex differences shown here. It is possible that in the latter time period, comparatively more females, who would otherwise have died before diagnosis, may have been diagnosed and treated, with consequent higher mortality seen in this group. Adjusted mortality was higher among females diagnosed and treated from 2012 to 2019 when compared to the 2008–2011 time period (data not shown), reinforcing this hypothesis. Males traditionally delay healthcare-seeking relative to females;^24^,^25^ this is also evident in South Africa.^26^ The recent South African TB prevalence survey highlighted that males were less likely to be diagnosed than their female counterparts, likely increasing their mortality risk.^27^ Post-mortem studies conducted in South Africa show that males with undiagnosed TB are over-represented.^28^,^29^ These data also support the male survivor effect hypothesis.

Challenges with treatment adherence might also contribute to higher early mortality among women. However, the literature suggests males struggle with adherence more than females as a result of work pressures and costs of treatment,^30^ while females might be less likely to be LTFU.^31^ Since direct adherence data were not available, we used loss to follow-up as a proxy for treatment adherence. By 6 months, we found significantly higher loss to followup in males than in females (7% vs. 4%). However, among patients who interrupted treatment, more females died before 6 months than males, again potentially suggesting a male survivor bias.

In many sub-Saharan settings, including South Africa, women are disproportionately impacted by the HIV epidemic.^32^ Indeed, females were more likely to be HIV-positive than males in our study. However, there was no difference in ART initiation or the proportion of HIV-positive individuals with CD4 counts <200 cells/mm^3^ between males and females. HIV infection is therefore an unlikely explanation for higher female mortality. The presence of other common comorbidities, such as diabetes mellitus (DM) and substance use disorder, could have had an impact on our mortality findings. In particular, the prevalence of DM is higher among women in our setting, and DM is a risk factor for TB/RR-TB mortality.^1^,^33^ Unfortunately, there were no reliable data available on other comorbidities to evaluate their potential impact on mortality in this study.

There are several other limitations in this study. First, this was a retrospective analysis of prospectively collected programmatic data conducted at a single site, which limits generalisability. Second, although we investigated multiple sources, including the deaths register, to determine vital status at 6-months post-treatment initiation, there was still a small number of patients for whom we lacked confirmation. Despite these limitations, we analysed data from a large, 11-year treatment cohort to assess mortality over time and the impact of programmatic changes.

## CONCLUSIONS

We found that treatment with LZD/BDQ/DLM reduced early mortality among patients who initiated treatment from 2012 to 2019, but not in the overall cohort. This is likely due to more individuals with MDR/RR-TB accessing treatment in more recent years, who would otherwise have died before diagnosis and treatment, in addition to initially restrictive criteria for access to LZD/BDQ/DLM. The former suggests that universal DST for all individuals with presumptive TB is necessary in all settings, while the latter suggests that scale-up of access to all-oral traetment regimens including newer drugs should be a priority. Increased early mortality among women paradoxically sugests that men may not be accessing TB diagnostic services, adding support to efforts to make healthcare services more accessible and patient-centred for all clients. Finally, these data highlight the need for further research investigating sex differences in accessing MDR/RRTB treatment.
